# A Personalized 14-3-3 Disease-Targeting Workflow Yields Repositioning Drug Candidates

**DOI:** 10.3390/cells14080559

**Published:** 2025-04-08

**Authors:** Yonika A. Larasati, Gonzalo P. Solis, Alexey Koval, Christian Korff, Vladimir L. Katanaev

**Affiliations:** 1 Translational Research Center in Oncohaematology, Department of Cell Physiology and Metabolism, Faculty of Medicine, University of Geneva, CH-1211 Geneva, Switzerland; yonika.larasati@unige.ch (Y.A.L.); gonzalo.solis@unige.ch (G.P.S.); alexey.koval@unige.ch (A.K.); 2 Pediatric Neurology Unit, University Hospitals of Geneva, CH-1211 Geneva, Switzerland; christian.korff@hcuge.ch; 3 Translational Oncology Research Center, Qatar Biomedical Research Institute (QBRI), College of Health and Life Sciences, Hamad Bin Khalifa University (HBKU), Qatar Foundation, Doha P.O. Box 34110, Qatar

**Keywords:** rare diseases, pediatric encephalopathy, *YWHAG*, 14-3-3γ, assay development, drug discovery, drug repositioning, phosphoproteins

## Abstract

Rare diseases typically evade the application of the standard drug discovery and development pipelines due to their understudied molecular etiology and the small market size. Herein, we report a rare disease-directed workflow that rapidly studies the molecular features of the disorder, establishes a high-throughput screening (HTS) platform, and conducts an HTS of thousands of approved drugs to identify and validate repositioning drug candidates. This study examines the pediatric neurological disorder caused by de novo mutations in *YWHAG*, the gene encoding the scaffolding protein 14-3-3γ, and the workflow discovers nuclear relocalization and a severe drop in 14-3-3γ binding to its phosphorylated protein partners as the key molecular features of the pathogenic hotspot *YWHAG* mutations. We further established a robust in vitro HTS platform and screened ca. 3000 approved drugs to identify the repositioning drug candidates that restore the deficient 14-3-3γ-phosphotarget interactions. Our workflow can be applied to other 14-3-3-related disorders and upscaled for many other rare diseases.

## 1. Introduction

**Figure 1:**
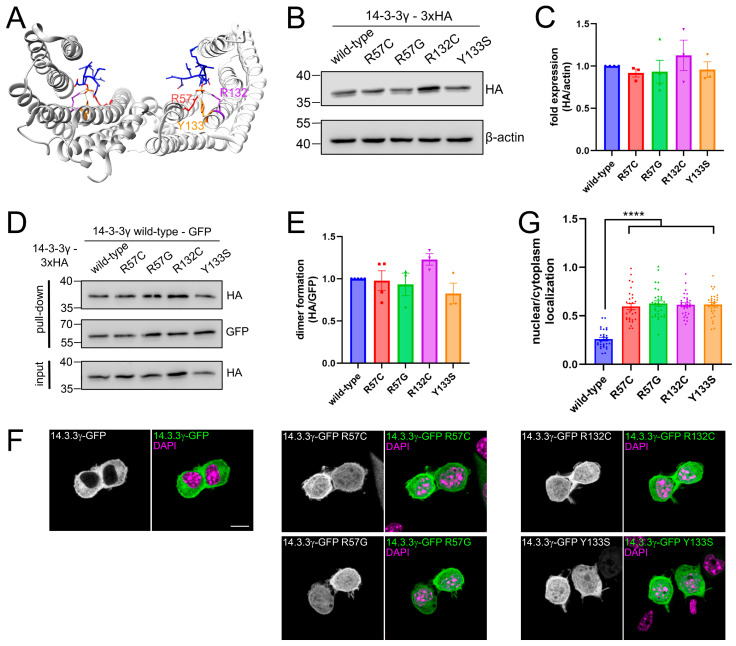
Cellular properties of pathogenic variants affecting residues R57, R132, and Y133 of 14-3-3γ**.** (**A**) Mutated amino acids (red, R57; purple, R132; orange, Y133) in the overall structure of the 14-3-3γ dimer complexed with the N-terminal (residues 1–43) phosphopeptide of tyrosine hydroxylase (PDB ID: 4J6S). These three residues are in the peptide binding groove and form hydrogen bonds with phosphopeptide (shown as a stick structure in blue). (**B**,**C**) Expression of C-terminally tagged wild-type and mutants 14-3-3γ. N2a cells were transfected with wild-type or mutants 14-3-3γ-3xHA. Protein expression was then analyzed by SDS–polyacrylamide gel electrophoresis (SDS-PAGE) and Western blot. Antibody against HA was used for detection of 14-3-3γ and β-actin was used as loading control (**B**). Quantification of the expression level of different 14-3-3γ constructs (**C**). (**D**,**E**) N2a cells were co-transfected with wild-type 14-3-3γ-GFP and mutants 14-3-3γ-3xHA. Immunoprecipitation (IP) of wild-type 14-3-3γ was performed using a nanobody against GFP, and the coprecipitation of mutants 14-3-3γ was analyzed by SDS-PAGE and Western blot (D). Antibody against GFP was used for the detection of wild-type 14-3-3γ and against HA for mutants 14-3-3γ. Quantification of the coimmunoprecipitation (co-IP) of mutants 14-3-3γ by wild-type 14-3-3γ (**E**). (**F**,**G**) Mutants R57C, R57G, R132C, and Y133S show aberrant nuclear localization. N2a cells expressing wild-type or mutants 14-3-3γ-GFP and stained with DAPI in magenta for nuclei. Scale bar: 10 μm (**F**). Mean fluorescence intensity ratios of 14-3-3γ-GFP variants at the nucleus versus cytoplasm (*n* = 28–30). Data in panels (**C**,**E**,**G**) represent mean ± SEM (*n* ≥ 3). Statistical analysis was performed using one sample *t*-test for panels C and E; and using one-way ANOVA for panel G followed by Dunnet’s multiple comparison test, **** *p* < 0.0001.

The 14-3-3 proteins are a family of evolutionarily conserved signaling proteins, with seven genes encoding different members in mammals: *YWHAB* (14-3-3β), *YWHAE* (14-3-3ε), *YWHAG* (14-3-3γ), *YWHAH* (14-3-3η), *YWHAQ* (14-3-3θ), *YWHAS* (14-3-3σ), and *YWHAZ* (14-3-3ζ) [1]. Most of them are expressed in the brain with distinct distributions and abundances [2]. While they do not possess enzymatic activities, 14-3-3 proteins act as adaptors by recognizing phosphoserine/threonine (pSer/pThr)-containing motifs of client proteins and controlling their properties, such as localization, activity, stability, or interaction with other proteins [1,3]. Structurally, 14-3-3 proteins exist as homo- or hetero-dimers with each monomer consisting of nine α-helices that are organized in an antiparallel array (Figure 1A). The dimer forms a cup-shaped space containing two phosphopeptide binding grooves [3]. With hundreds of identified client partners, the 14-3-3 proteins have been implicated in various diseases, including cancer, neurological conditions, and reproductive disorders [4].

The *YWHAG* gene encoding 14-3-3γ, located in the 7q11.23 chromosomal region, has emerged as a significant contributor to rare neurodevelopmental disorders, particularly those associated with epilepsy and intellectual disabilities [5]. Starting from 2010, the first chromosomal deletions covering the gene *YWHAG*, and later truncating and missense mutations in *YWHAG* have been reported to cause a developmental epileptic encephalopathy affecting infants and children (DEE56, OMIM#617665). Upon further clinical investigations, *YWHAG*-related disorders have been found to exhibit a wide spectrum of neurodevelopmental problems, mostly characterized by early-onset epilepsy, intellectual disability, motor developmental delay, speech impairment, and behavioral problems [5,6,7,8,9,10,11]. At least 20 pathogenic variants affecting 14-3-3γ have been reported, with around 50 patients identified worldwide [8,12]. Heterozygous mutations affecting *YWHAG* are mostly de novo; however, inherited mutations have also been reported in small fractions of patients. Intriguingly, amino acids of the conserved triad Arg57-Arg132-Tyr133 (R57-R132-Y133) within the phosphopeptide-binding groove of 14-3-3γ (Figure 1A) are responsible for >50% of *YWHAG*-related disorders cases and lead to the most severe clinical phenotypes [6,7,8,13].

In the current study, we extensively characterized the most common pathogenic variants of 14-3-3γ: R57C, R57G, R132C, and Y133S [8,12]. We describe the molecular defects of these pathogenic variants, including their loss of interaction with partner phosphopeptides (in vitro) and phosphoproteins (in cells). Based on these data, we then established a robust high-throughput screening (HTS) platform to find drug repositioning candidates correcting the capability of mutant 14-3-3γ to bind phosphopeptides. The identified and validated hits may find their swift way toward novel treatments for this rare yet devastating neurological disorder, and our approach can be applied to other 14-3-3-related diseases.

## 2. Materials and Methods

### 2.1. Peptides

The following peptides were synthesized by AbClonal (Woburn, MA, USA) (Table 1). Phosphopeptides (corresponding to the respective human sequences) were labeled with FITC in their N-terminus.

### 2.2. Cloning and Plasmid Constructs

The plasmid encoding GST-tagged wild-type human 14-3-3γ was purchased from Addgene (Watertown, MA, USA) (#109905) [18] and was used to subclone the wild-type sequence into the following vectors using the following primer pairs and the Gibson assembly method according to the manufacturer’s instructions (NEBuilder HiFi Mix, E2621S; NEB, Ipswich, MA, USA) to obtain the plasmids listed in Table 2.

Plasmid p3xHA-N1 was derived from pEGFP-N1 by seamless replacement of the EGFP ORF with that of 3 consecutive HA tags. Subsequently, mutants for each of these plasmids were generated using a site-directed mutagenesis approach with KOD polymerase (Sigma-Aldrich, St. Louis, MO, USA, KMM-201NV) followed by DpnI digestion of the template.

The human tyrosine hydroxylase (TH) sequence (SinoBiological, Beijing, China, HG10684-M) was amplified by PCR and cloned in frame into the BspEI/BglII sites of the p3xHA-C1 plasmid [21] to generate 3xHA-TH construct. To generate GFP-SLP76 construct, the human SLP76 sequence (SinoBiological, HG11237-M) was amplified by PCR and cloned in frame into the EcoRI/BamHI sites of pEGFP-N1 [21].

The primers used to generate the constructs encoding the mutants 14-3-3γ, 3xHA-TH, and GFP-SLP76 are listed in Table 3.

### 2.3. Cell Culture

Mouse neuroblastoma Neuro-2a (N2a; CCL-131; ATCC) cells were maintained in MEM (Thermo Fisher Scientific, Waltham, MA, USA), supplemented with 10% FCS, 2 mM L-glutamine, 1 mM pyruvate, and 1% penicillin–streptomycin at 37 °C and 5% CO_2_. Human HEK293T (CRL-3216, ATCC) cells were grown in DMEM (Thermo Fisher Scientific), supplemented as above. Human SH-SY5Y cells were kindly given by Dr. Annick Mühlethaler-Motte (University Hospital of Lausanne) and were grown in DMEM low-glucose (Thermo Fisher Scientific), supplemented as above.

### 2.4. Expression of 14-3-3γ in N2a Cell

For 14-3-3γ expression analysis, N2a cells were seeded on 48-well culture plates (5 × 10^4^ cells/well) and 24 h later were transfected with 0.3 μg of wild-type or mutants 14-3-3γ-GFP/14-3-3γ-3xHA. After an additional 24 h, the cells were harvested with RIPA buffer (150 mM NaCl, 1% Triton X-100, 0.5% sodium deoxycholate, 0.1% sodium dodecyl sulfate (SDS), and 50 mM Tris). After centrifugation, the supernatants were analyzed by Western blotting using antibodies against GFP (Genetex, Irvine, CA, USA, GTX113617) or HA (Abcam, Cambridge, UK, ab9110) and β-actin (Proteintech, Rosemont, IL, USA, 81115-1-RR) as loading control.

### 2.5. Coimmunoprecipitation Assays

N2a or HEK293T cells (4 × 10^5^ cells/well) were seeded on 6-well plates and cultured for 24 h before co-transfection with 3 μg total DNA using the following combinations: wild-type 14-3-3γ-GFP and wild-type/mutant 14-3-3γ-3xHA (1:1), wild-type 14-3-3γ-GFP and 3xHA-TH (1:2), or GFP-SLP76 and 14-3-3γ-3xHA (2:1). After 24 h of transfection, the cells were resuspended in ice-cold lysis buffer (50 mM Tris-HCl, pH 8.0, 150 mM NaCl, 0.6% Triton X-100, and 0.2% glycerol) supplemented with a protease inhibitor cocktail and a phosphatase inhibitor cocktail (both were from Roche) then passed >10 times through a 25 G needle. Extracts were cleared by centrifugation at 15,000× *g* for 15 min at 4 °C, and supernatants were incubated with 2 μg of purified GST-tagged GFP-nanobody [22] for 30 min on ice. Then, 20 μL of Glutathione Sepharose 4B beads were added, samples were rotated at 4 °C overnight (for 14-3-3γ dimer) or for 3 h (binding partner interaction), beads were repeatedly washed with lysis buffer, prepared for SDS-PAGE, and finally analyzed by Western blot using antibodies against GFP and HA.

### 2.6. Immunofluorescence

N2a or SH-SY5Y cells (1.5 × 10^5^ cells/well) were seeded on 12-well culture plates. The next day, the cells were transfected with 0.5 μg of wild-type or mutants 14-3-3γ-GFP/14-3-3γ-3xHA for 7 h, trypsinized and seeded on poly-L-lysine-coated coverslips in complete MEM for additional 15–17 h before fixation. Cells were fixed with 4% paraformaldehyde in PBS for 20 min, permeabilized for 1 min using ice-cold PBS supplemented with 0.1% Triton X-100, and blocked for 30 min with PBS supplemented with 1% BSA. Cells transfected with the 14-3-3γ-3xHA constructs were incubated with anti-HA-tag (Roche, Basel, Switzerland; 1/500) in blocking buffer for 2 h at room temperature (RT), washed, and then incubated with the secondary anti-Rat Cy3-conjugated (Jackson ImmunoResearch, West Grove, FL, USA; 1/500) and DAPI also in a blocking buffer for 2 h at RT. Cells transfected with 14-3-3γ-GFP variants were only stained with DAPI. Finally, cells were mounted with VECTASHIELD on microscope slides, and recorded with a Plan-Apochromat 63x/1.4 oil objective on a LSM800 Confocal Microscope using the ZEN 2.3 software (all Zeiss, Oberkochen, Germany). Mean fluorescence intensity was determined from confocal images using ImageJ v1.54f (National Institutes of Health), and ratio fluorescence values were used for quantification as previously reported [23].

### 2.7. Recombinant Protein Production and Purification

*E. coli* strain Rosetta(DE3)pLysS (Novagen, Madison, WI, USA, 70956) was transformed with pet23b-His_6_-nLuc or pet23b-His_6_-14-3-3γ-nLuc (wild-type or mutants) and grown at 37 °C to an OD_600_ = 0.6 before induction with 0.25 mM IPTG and additional growth overnight at 18 °C. Bacteria were then harvested by centrifugation 3500× *g* at 4 °C and resuspended in TBS (20 mM Tris-HCl (pH 7.5) and 150 mM NaCl) supplemented with 1 mM PMSF and 30 mM imidazole (all from Sigma-Aldrich). Cell lysis was performed using a French pressure cell press and lysate was cleared by centrifugation at 15,000× *g* for 15 min at 4 °C. Supernatants were incubated overnight with Ni-NTA Agarose beads (QIAGEN, Hilden, Germany) in a rotary shaker at 4 °C. The beads were washed five times with 10 resin volumes of ice-cold wash buffer (TBS supplemented with 10 mM imidazole). Proteins were finally eluted with TBS containing 300 mM imidazole. Imidazole was removed by buffer exchange to TBS using Vivaspin Centrifugal concentrators. Protein concentration was measured using the Bradford assay, and the purity was analyzed using SDS-PAGE followed by Coomassie staining.

### 2.8. In Vitro BRET Assay

Recombinant nLuc or 14-3-3γ-nLuc (final concentration 1 nM) and various concentrations of FITC-labeled phosphopeptides were diluted in the reaction buffer (20 mM Tris-HCl, 150 mM NaCl, 1 mg/mL bovine serum albumin (BSA), and 0.1% Tween 20). The mixture was then pipetted into black 384-well plates (Greiner, Kremsmünster, Austria), and furimazine (250 nM; Chemshuttle, Burlingame, USA) was added into the wells. FITC and nLuc signals were read using the built-in NanoBRET filter system in a Tecan Infinite M200 PRO plate reader, and ratios of FITC/nLuc signal was calculated as BRET signal. BRET signal from nLuc + FITC-phosphopeptides (background BRET) was used to subtract BRET signal from 14-3-3γ-nLuc + FITC-phosphopeptides to obtain net BRET. Kd for each phosphopeptide was calculated using GraphPad Prism v.10.4.0 using the formula y = Bmax*x/(Kd + x). To calculate Kd of phosphopeptides to 14-3-3γ-R57C-nLuc, Bmax was set to that of wt 14-3-3γ-nLuc.

### 2.9. High-Throughput Screening and Hit Validation

HTS for mutant 14-3-3γ-nLuc modulators was performed using the 14-3-3γ-R57C-nLuc protein, FITC-ppLRRK2, and FDA Approved and Pharmacopeial Drug Library (HY-L066, MedChemExpress, Monmouth Junction, NJ, USA). Dimethyl sulfoxide (DMSO) or compounds in DMSO (12.5 μM) were mixed with 14-3-3γ-R57C-nLuc (1nM) and FITC-ppLRRK2 (100 nM) in a reaction buffer and furimazine (250 nM) was added before BRET measurement as described in the “in vitro BRET assay” section above. Z’ factor for each plate was calculated according to [24]. Primary hits were picked if the compound modulated the BRET signal ± 3SD. The hits were subsequently validated by performing the BRET assay at 12.5 μM of compounds (in triplicate) using 14-3-3γ-R57C-nLuc, wild-type 14-3-3γ-nLuc, or nLuc and FITC-LRRK2. Validated hits were purchased from MedChemExpress: nafamostat mesylate (HY-B0190A) and its analog sepimostat (HY-136299-S), degarelix (HY-16168A), aviptadil acetate (HY-P0012A), and oritavancin diphosphate (HY-B1831A).

## 3. Results

### 3.1. Mutations Affecting R57, R132, and Y133 Display Normal Cellular Expressions but Aberrant Localizations

We first evaluated the cellular properties of pathogenic variants R57C, R57G, R132C, and Y133S affecting the phosphopeptide-binding triad of 14-3-3γ in N2a cells, a murine neuroblastoma cell line routinely used in neurodevelopmental studies [21,22,25]. Previous studies have reported that the use of either a small tag such as His_6_ or a bigger tag such as GST or YFP does not affect 14-3-3 protein expression, dimerization, and subcellular localization [26,27,28]. We found that wild-type 14-3-3γ with a GFP tag in either N- or C-terminal displayed similar expression levels and cellular localization (Figure S1A,B). We then evaluated further cellular properties using the C-terminally tagged 14-3-3γ, by either GFP or 3xHA tags. We found that the pathogenic variants R57C, R57G, R132C, and Y133S were well-expressed in N2a cells: to levels similar to the wild-type (wt) protein in the case of the 14-3-3γ-3xHA fusions (Figure 1B,C), and with the levels slightly reduced in the case of 14-3-3γ-GFP fusions (Figure S1C,D).

Through the interaction of N-terminal helices of the 14-3-3 proteins, homo- and/or hetero-dimers can be formed [29]. In 14-3-3γ, residue R57 is localized in helix 3, while R132 and Y133 are in helix 5, neither of which contribute to the dimer formation. Therefore, we did not expect that the mutations affecting those residues affect the dimerization of 14-3-3γ. To confirm this hypothesis, we performed co-immunoprecipitations (co-IPs) assay using wt 14-3-3γ-GFP and wt or mutant 14-3-3γ-3xHA in N2a cells (Figure 1D). Indeed, the four pathogenic 14-3-3γ-3xHA variants were equal to wt in the binding to wt 14-3-3γ-GFP (Figure 1D,E).

We then evaluated the localization of wt and mutant 14-3-3γ in N2a cells. We found that wt 14-3-3γ with either GFP (Figure 1F) or HA tags (Figure S1E) localized in the cytoplasm, in line with a previous study [30]. Interestingly, all pathogenic variants exhibited aberrant localizations, diffusing into the nucleus in addition to their cytoplasmic localization (Figure 1F). Quantification of the average fluorescence intensities of the nuclear and cytoplasmic signals showed a nucleus/cytoplasm ratio of ca. 0.26 for wt 14-3-3γ-GFP, the value increasing >2-fold for the four pathogenic variants (Figure 1G). This increase in the nuclear localization was accompanied by a slight but measurable (ca. 15%) decrease in the cytoplasmic signal of the four pathogenic variants (Figure S1F). As this phenotype was also observed in the case of the 14-3-3γ-3xHA fusions (Figure S1E), it cannot be attributed to the bulky GFP fusion and reflects the natural mislocalization of the pathogenic variants. The increased nuclear localization of 14-3-3γ-R57C was also observed in human neuroblastoma SH-S5Y5 cells (Figure S1G,H).

### 3.2. Mutations Affecting R57, R132, and Y133 Fail to Interact with the Binding Partners In Vitro and in Cells

Several proteins have been shown to interact with 14-3-3γ, with tyrosine hydroxylase (TH) and leucine-rich repeat kinase 2 (LRRK2) as the most well-studied neuronal partners [14,16,31,32]. TH is a rate-limiting enzyme in the synthesis of dopamine and other catecholamine neurotransmitter, in which TH catalyzes the hydroxylation of tyrosine to L-DOPA [33]. LRRK2 is a large and multifunctional serine/threonine-protein kinase, and mutations in its gene are the most common genetic predisposition for Parkinson’s disease [34]. Among 14-3-3 family members, 14-3-3γ is the strongest binder and activator of TH [14,35], and it is also the strongest binder for LRRK2-derived phosphopeptides [36]. Another validated binding partner of 14-3-3γ is SLP76 (also known as Lymphocyte cytosolic protein 2/LCP2), found in T-cells and related lymphocytes [15].

**Figure 2:**
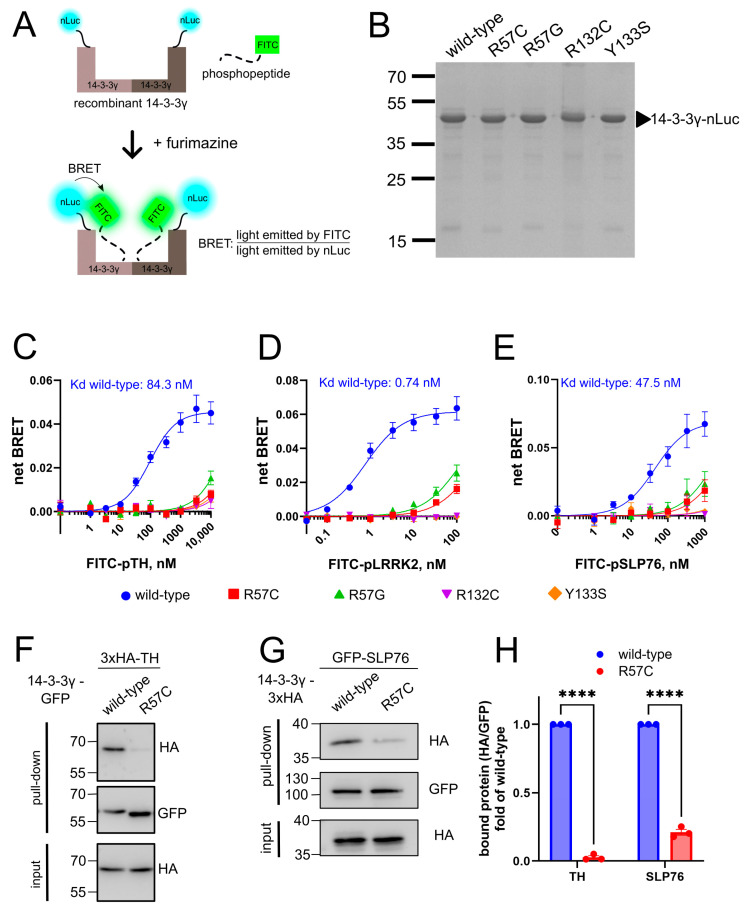
Mutants R57C, R57G, R132C, and Y133S lose interactions with partner phosphoprotein in vitro and in cells. (**A**) A novel in vitro BRET assay developed in this study to assess the interaction between recombinant 14-3-3γ (C-terminally tagged with nanoluc (nLuc; BRET donor)) and partner phosphopeptide (N-terminally tagged with FITC; BRET acceptor). Upon the addition of furimazine, substrate for nLuc, light emitted by nLuc excites FITC in the close proximity (<10 nm), which occurs when FITC-phosphopeptide bound to 14-3-3γ-nLuc. (**B**) The production and purification of recombinant 14-3-3γ-nLuc from *E. coli* showed similar expression levels and purity between wild-type and mutants 14-3-3γ-nLuc. (**C**–**E**) Recombinant 14-3-3γ-nLuc (1 nM) was mixed with increasing doses of FITC-pTH (**C**), FITC-ppLRRK2 (**D**), or FITC-pSLP76 (**E**) before the addition of 250 nM furimazine. Wild-type 14-3-3γ-nLuc exhibits increased BRET signal in a dose-dependent manner, while mutants 14-3-3γ-nLuc show significantly decreased BRET signals indicating their incapability to efficiently bind FITC-phosphopeptides. (**F**–**H**) Cellular interactions of 14-3-3γ with TH or SLP76 as determined by IP. N2a cells were co-transfected with wild-type or R57C mutant 14-3-3γ-GFP and 3xHA-TH. IP of 14-3-3γ was performed using a nanobody against GFP, and the co-precipitation of TH was analyzed by SDS-PAGE and Western blot. Antibody against GFP was used for the detection of 14-3-3γ and against HA for TH (**F**). N2a cells were co-transfected with GFP-SLP76 and wild-type or R57C mutant 14-3-3γ-3xHA. IP of SLP76 was performed using a nanobody against GFP, and the co-precipitation of 14-3-3γ was analyzed by SDS-PAGE and Western blot. Antibody against GFP was used for the detection of SLP76 and against HA for 14-3-3γ (**G**). Quantification of the coimmunoprecipitation between 14-3-3γ and TH or SLP76 (**H**). Data in the panels (**C**–**E**,**H**) represent mean ± SEM (*n* ≥ 3). Statistical analysis for panel H was performed using one sample *t*-test, **** *p* < 0.0001.

R57, R132, and Y133 are the triad residues essential for the binding of 14-3-3γ to the phosphoserine/phosphothreonine (pSer/pThr) motif of the client binding partners [13]. Therefore, mutations affecting those residues have been hypothesized to diminish the ability of 14-3-3γ to bind phosphoproteins [6,7]. To experimentally test this hypothesis, we established a novel in vitro bioluminescence resonance energy transfer (BRET) assay using recombinant 14-3-3γ tagged with nanoluciferase (14-3-3γ-nLuc) and phosphopeptides labeled with the FITC fluorophore (FITC-phosphopeptides) (Figure 2A). Upon the addition of furimazine (substrate for nLuc), 14-3-3γ-nLuc acts as a BRET donor and excites FITC-phosphopeptides in its close proximity (<10 nm). The BRET signal is defined as the fluorescence emitted by FITC-phosphopeptides divided by the luminescence emitted by 14-3-3γ-nLuc.

We successfully produced the recombinant 14-3-3γ-nLuc wt in *E. coli* (size ~47.5 kDa), with high yield (~3.5 mg protein/L culture) and purity (>95%). Pathogenic 14-3-3γ variants were produced and purified as nLuc fusions in yield and purity equal to wt (Figure 2B), alongside with recombinant nLuc that was used for subtraction of the background BRET signal. We then evaluated the ability of wt and mutant 14-3-3γ-nLuc to bind FITC-labeled phosphopeptides derived from TH (FITC-pTH), SLP76 (FITC-pSLP76), and LRRK2 (FITC-ppLRRK2 harboring two pSer residues, see Section 2). 14-3-3γ-nLuc wt elicited a strong and dose-dependent BRET signal in the presence of phosphopeptides, a phenomenon not observed for the nLuc control (Figure S2A–C).

The net BRET signal was then calculated by subtracting the background nLuc signal from that of 14-3-3γ-nLuc. With increasing concentrations of all three tested phosphopeptides, an increase in the BRET signal was observed for wt 14-3-3γ-nLuc (Figure 2C–E). The resulting dose saturation curves permitted us to calculate the binding constants (K_d_) for the 14-3-3γ—phosphopeptide interactions as 84.3 nM (pTH), 0.74 nM (ppLRRK2), and 47.5 nM (pSLP76). The highest affinity of ppLRRK2 was determined by the dual phosphorylation of this peptide. Interestingly, our K_d_ values are lower than those obtained previously by other methods (550 nM, 2 nM, and 390 nM, respectively [14,15,16]), suggesting the higher sensitivity of this assay to monitor the interaction between 14-3-3γ and client phosphopeptides.

Using this novel in vitro BRET assay, we next proved that pathogenic 14-3-3γ variants R57C/G, R132C, and Y133S have the strongly reduced ability to bind all three phosphopeptides (Figure 2C–E). Of note, ca. 30% of the phosphopeptide binding could still be reached by the variants R57C and R57G at the highest concentrations of FITC-ppLRRK2 and -pSLP76 (Figure 2D,E). Meanwhile, the variants R132C and Y133S appeared to totally lose their capacities to bind the phosphopeptides (Figure 2D,E).

Next, we also evaluated the responsiveness of our in vitro BRET assay towards external stimuli using R18, a peptide well-characterized as an inhibitor of 14-3-3 interaction with binding partners [17]. As expected, R18 inhibited the binding of all three phosphopeptides to wt 14-3-3γ-nLuc in a dose-dependent manner (Figure S2D), indicating that our assay can be used to evaluate potential modulators of 14-3-3γ—phosphopeptide interactions. Another compound tested was fusicoccin A, a molecular glue known to increase the interaction of several members of the 14-3-3 protein family with their binding partners [37]. However, we did not observe an increased affinity of pTH (Figure S2E) or ppLRRK2 (Figure S2F) towards 14-3-3γ-nLuc, wt or R57C, in the presence of 100 μM fusicoccin A. Our result is in agreement with previous data showing that fusicoccin A, unlike its effects on other 14-3-3 family members, does not enhance the binding of 14-3-3γ to various pLRRK2 peptides [16], and showed only a minor effect for a CAMKII-derived phosphopeptide [37].

To confirm the results from the in vitro BRET assay, we performed co-IPs using nanobody against GFP to evaluate the ability of mutant 14-3-3γ-R57C to interact with partner proteins in the natural environment of N2a cells. First, we performed a co-IP with 14-3-3γ-GFP and 3xHA-TH. While wt 14-3-3γ efficiently co-precipitated TH, 14-3-3γ-R57C failed to bind TH (Figure 2F,H). We then performed a co-IP with GFP-SLP76 and 14-3-3γ-3xHA, producing a similar observation (Figure 2G,H). Decreased interactions between 14-3-3γ-R57C and TH or SLP76 were also observed in human HEK293T cells (Figure S2G,H). Altogether, these data align with our in vitro findings and prove the hypothesis that the 14-3-3γ pathogenic variants R57C/G, R132C, and Y133S are strongly impaired in their capacity to interact with its partners.

### 3.3. High-Throughput Screening to Find Repositioning Drug Candidates Restoring the Phosphopeptide Binding by Pathogenic 14-3-3γ Variants

**Figure 3:**
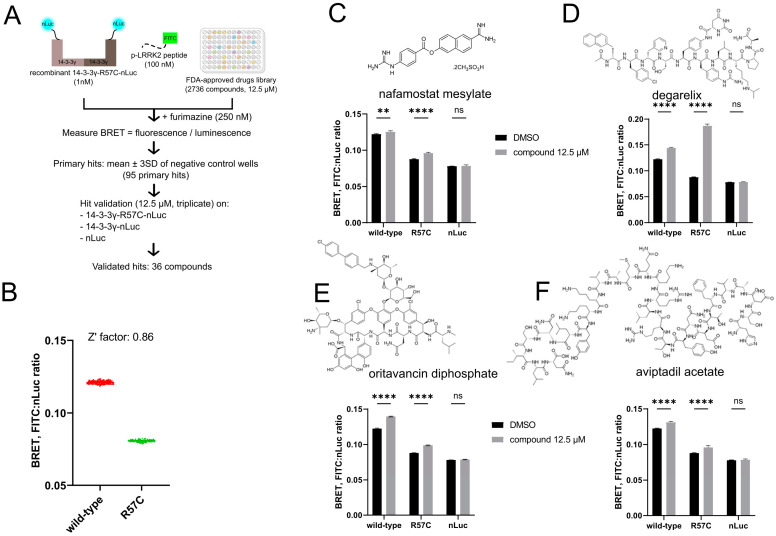
High-throughput screening (HTS) to find a modulator of 14-3-3γ-R57C with pLRRK2. (**A**) HTS pipeline using the established in vitro BRET assay and FDA-approved drugs and pharmacopeial library. (**B**) In the 384-wellplate format, the positive control (wild-type 14-3-3γ-nLuc + FITC-ppLRRK2) and negative control (14-3-3γ-R57C-nLuc + FITC-ppLRRK2) exhibited a wide separation window with a calculated Z’ factor of 0.86, indicating a robust assay for HTS. (**C**–**F**) Four hits increasing BRET signal were validated in 14-3-3γ-R57C-nLuc, wild-type 14-3-3γ-nLuc, and nLuc, which are nafamostat mesylate (**C**), degarelix (**D**), oritavancin diphosphate (**E**), and aviptadil acetate (**F**). Data in (**C**–**F**) are mean ± SEM (*n* = 3). Statistical analysis was performed using two-way ANOVA followed by Sidak test. ns, not significant; **, *p* < 0.01; ****, *p* < 0.0001.

We confirmed the diminished ability of the pathogenic 14-3-3γ variants to interact with the binding partners in vitro and in vivo—the feature that likely lies at the molecular core of the pathology of *YWHAG*-related disorders. Identifying a small molecule that could restore the deficient interaction could lead to targeted therapy for this encephalopathy. Following our recent example of such an approach dedicated to another rare disease—the *GNAO1* encephalopathy [19,20,25]—we built a high-throughput screening (HTS) pipeline using our in vitro BRET assay to look for a repositioning small molecule drug candidate that could modulate the interaction between mutant 14-3-3γ and a phosphopeptide (Figure 3A). We used 14-3-3γ-R57C-nLuc as the hotspot *YWHAG* mutation [12] and FITC-ppLRRK2 for the HTS as this peptide showed the highest affinity towards 14-3-3γ (Figure 2D).

For the HTS, the BRET signal from wt 14-3-3γ-nLuc and FITC-ppLRRK2 was defined as the positive control, while the BRET signal from the R57C variant and FITC-ppLRRK2 was defined as the negative control (Figure 3B). The separation window between the positive and negative controls of the HTS should be wide enough to produce a robust screening and avoid false positives/negatives. The calculation of the Z’ factor is a helpful tool to evaluate the robustness of the assay, with values between 0.5 and 1 considered excellent, values between 0 and 0.5—acceptable, and values less than 0 indicating that the assay is unlikely to function in a high-throughput context [24]. The calculated Z’ factor for our HTS in a 384-wellplate format was 0.86, highlighting that we have established an assay that is excellent for HTS (Figure 3B).

We then used this assay to screen a library of 2736 US FDA-approved and pharmacopeial drugs (Figure 3A). The primary hit criterion was the modulation of the BRET signal by >±3SD of the negative control. HTS analysis after the screening confirmed the high robustness with the calculated Z’ factors across 8 HTS plates ranging from 0.73 to 0.84 (Figure S3). From this primary screening, 95 hits were obtained and re-screened against 14-3-3γ-R57C-nLuc in triplicates, validating 36 compounds. We further tested them on wt 14-3-3γ-nLuc to analyze the possible selectivity of the compounds towards the mutant protein, as well as on nLuc to eliminate compounds affecting the general luminescence of nLuc (Figure 3A). Resultingly, we found that 11 compounds affected nLuc, 4 compounds decreased the BRET signal of wt only, 17 compounds decreased the BRET signal of both mutant and wt, and 4 compounds increased the BRET signal of both mutant and wt. These four hits that increased the BRET signal of both R57C and wt 14-3-3γ were as follows: nafamostat mesylate, degarelix, oritavancin diphosphate, and aviptadil acetate (Figure 3C–F). Among them, we prioritized nafamostat mesylate and degarelix for a more detailed examination as the first was the only non-peptide drug of the four, and the second showed a more prominent effect in increasing the BRET signal for the mutant R57C than wt 14-3-3γ (Figure 3C,D).

We tested the effect of nafamostat mesylate and degarelix in a dose-dependent manner using 1 nM of nLuc or 14-3-3γ-nLuc (wt or R57C) and 100 nM of FITC-ppLRRK2 (Figure S4A,B). Neither compound affected the BRET signal of nLuc and FITC-ppLRRK2 even at 100 μM (Figure S4A,B; blue curves). Nafamostat mesylate started to increase the BRET signal for 14-3-3γ-R57C-nLuc at 3.3 μM and, remarkably, reached the signal equal to that of wt 14-3-3γ-nLuc at 33 μM (Figure S4A). At this concentration, the drug also modestly but significantly increased the BRET signal of wt 14-3-3γ-nLuc (Figure S4A).

As for degarelix, the compound significantly increased the BRET signal of 14-3-3γ-R57C-nLuc and FITC-ppLRRK2 in all doses tested, with the highest BRET signal achieved at 1 μM of the compound, and the effect becoming less pronounced but still significant at higher concentrations (Figure S4B). This peculiar dose dependence likely resulted from the known capacity of this peptide to aggregate at high concentrations, limiting its effective concentration [38].

**Figure 4:**
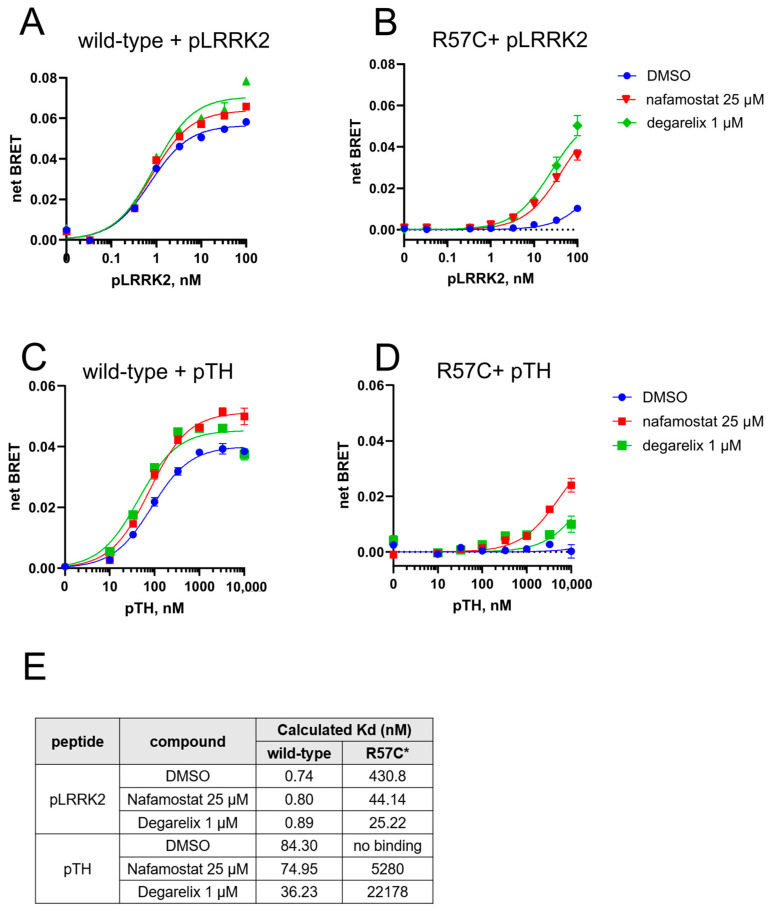
In vitro validation of nafamostat and degarelix as modulators of 14-3-3γ-nLuc and phosphopeptides interaction. (**A**–**D**) Recombinant 14-3-3γ-nLuc (1 nM) was mixed with increasing concentrations of FITC-ppLRRK2 (**A**,**B**) or FITC-pTH (**C**,**D**) in the presence of 25 μM nafamostat or 1 μM degarelix before subjected to in vitro BRET assay. (**E**) Calculated Kd for both phosphopeptides toward wild-type 14-3-3γ-nLuc or 14-3-3γ-R57C-nLuc in the presence of drugs. Due to the lack of binding between 14-3-3γ-R57C-nLuc and phosphopeptides, its Bmax is set to Bmax of wild-type 14-3-3γ-nLuc using the same phosphopeptide. Nafamostat mesylate and degarelix increase the binding of phosphopeptides (decrease calculated Kd) towards 14-3-3γ-R57C-nLuc. Data are mean ± SEM (*n* = 4–8). * indicates that apparent Kd of 14-3-3γ-R57C-nLuc were calculated, with Bmax set to that of wt 14-3-3γ-nLuc.

Based on these results, we used 25 μM nafamostat mesylate and 1 μM degarelix to evaluate their effect on the affinity of phosphopeptides towards 14-3-3γ-nLuc, wt or R57C (Figure 4A–D) and to calculate the K_d_ of the phosphopeptide—14-3-3γ (wt and R57C variants) interactions, in the presence and absence of the drugs (see Section 2 for details). Interestingly, while both nafamostat mesylate and degarelix could increase the maximum BRET signal achieved upon the wt protein interaction with ppLRRK2, the affinity of this interaction was not affected by the drugs (Figure 4A,E). In contrast, both the maximum BRET signals and the affinities of the R57C-ppLRRK2 interaction were strongly improved by nafamostat mesylate and degarelix, with the BRET signals increasing by 3 to 5-folds (Figure 4B), and the affinities—by 10 to 16-folds (Figure 4E). These findings reveal a strong and pathogenic variant-selective effect of nafamostat mesylate and degarelix on the restoration of the phosphopeptide binding. We also tested the orally available analog of nafamostat mesylate, sepimostat [39], which proved less efficient in restoring the interaction between 14-3-3γ[R57C] and ppLRRK2 (ca. 1.5 folds at 25 µM, Figure S4C,D). The two other hits from HTS, aviptadil acetate and oritavancin diphosphate, were also tested (at 25 μM) for their effects on ppLRRK2 binding towards 14-3-3γ-nLuc, wt or R57C. Both were also found to increase the maximum BRET signal of the two 14-3-3γ-nLuc variants (Figure S4E,F), although the effects were less prominent for the R57C mutant (ca. 2-folds) compared to nafamostat mesylate and degarelix.

We next validated nafamostat mesylate and degarelix on another phosphopeptide, pTH. Both drugs increased the affinity of pTH towards 14-3-3γ-nLuc wt (Figure 4C,E). The R57C pathogenic variant, which did not bind pTH even at the highest concentration of the peptide (Figure 2C), now exhibited measurable binding to pTH in the presence of either compound (Figure 4D,E).

Altogether, we successfully developed a novel and robust BRET-based HTS pipeline to find small molecules capable of modulating the interaction between 14-3-3γ and partner phosphopeptides—resulting in the discovery of four candidate repositioned drugs that increased the interaction between phosphopeptides and pathogenic 14-3-3γ.

## 4. Discussion

Increasing access to genetic testing has enabled the identification of the genetic etiology in ~50% of DEE cases [40], opening the possibility for the development of personalized medicines to improve the treatment and prognosis of patients affected by these devastating diseases. We have previously applied this concept to *GNAO1* encephalopathy [20,25,41], a broad-spectrum neurological disorder caused by a dominant heterozygous mutation in *GNAO1*—the gene encoding the major neuronal G protein Gαo. We established biochemical, cellular, patient iPS-based, and animal models of the disease and identified the underlying dominant missense mutations as neomorphs (as opposed to loss-of-function (LOF), dominant negative, or gain-of-function (GOF)) [19,22,25,42,43,44,45]. Using these tools, we developed a pipeline of personalized drug discovery that led to the discovery of zinc salts as a safe treatment for patients with a subset of *GNAO1* mutations [20,25], currently in clinical trials (registered: NCT06412653).

Herein, we applied our rare disease-directed drug discovery workflow [41] to the *YWHAG*-related disorders, another group of monogenic neurodevelopmental disorders with a wide spectrum of phenotypes ranging from mild developmental delay to DEE. With an increasing number of patients bearing *YWHAG* variants, a genotype–phenotype relationship for this disease starts to emerge. A careful comparison of clinical studies classifies the pathogenic variants into three groups: (1) frameshift or nonsense mutation resulting in truncated protein variants, (2) missense mutations outside the phosphopeptide binding groove, and (3) missense mutations inside the phosphopeptide binding groove [8]. Interestingly, none of the truncated variants (first group) result in DEE; on the other hand, mutations within the peptide binding groove lead to the most severe clinical manifestations [8]. Large analysis of the patient treatment responses also indicates that, although not statistically significant with the current small number of patients, good seizure control is more often achieved for truncated variants and mutations outside the peptide binding groove (groups 1 and 2) than for the most clinically severe group 3 mutations [8]. Thus, the currently available clinical data highlight the hotspot mutations within the phosphopeptide binding groove of 14-3-3γ as the most clinically severe, urging for the comprehensive molecular and cellular assessment of these pathogenic variants and their mechanisms of pathogenicity.

While it had been hypothesized that mutations in the phosphopeptide binding groove would result in the loss of interaction between 14-3-3γ and partner phosphoproteins [7,8], we provide the first experimental evidence confirming that indeed, mutations in the residues R57, R132, and Y133 result in the severe drop of 14-3-3γ interaction with various phosphoproteins in vitro and in cells. Of those, TH is regulated by phosphorylation in multiple sites (i.e., Thr8, Ser19, Ser 31, and Ser40) that alter its enzymatic kinetics [33,46]. Although 14-3-3γ has been shown as the strongest activator of TH in a pSer19-dependent manner [35], the functional effects of the mutants 14-3-3γ studied here on the activity of TH and catecholamine neurotransmitter’s levels remain to be evaluated. LRRK2 is a large 286 kDa protein consisting of seven domains, among them are a catalytic core with a GTPase and kinase domain [47]. LRRK2 has been reported to be involved in cytoskeleton and membrane trafficking, as well as driving neurite formation [48]. The binding of 14-3-3γ to LRRK2 has been shown to regulate LRRK2’s cellular localization and kinase activity [47,49,50]. Previous studies demonstrate that multiple PD-related LRRK2 mutants lose their interactions with 14-3-3γ, resulting in the accumulation of LRRK2 within cytoplasmic puncta that resemble inclusion bodies with hyper-ubiquitination of the protein [50,51]. Similarly to TH, the functional consequences of pathogenic 14-3-3γ mutations on cellular LRRK2 will require further investigations.

As 14-3-3 proteins homo- and hetero-dimerize to exert their activities, and since *YWHAG*-related disorders are caused by heterozygous dominant mutations, an important question to understand the disease etiology is whether the pathogenic variants maintain their capacity to dimerize and form dimers with the wt form of 14-3-3γ. Our findings unequivocally show that the four severe missense mutations affecting the phosphopeptide-binding groove, R57C, R57G, R132C, and Y133S, are as efficient in forming dimers with 14-3-3γ wt as the wt form itself. Thus, it is reasonable to expect that these pathogenic variants are not merely LOF/haploinsufficient (which is also supported by the fact that clear LOF mutations in *YWHAG* produce milder clinical phenotypes), but dominant negative in their genetic mechanism.

An interesting cellular phenotype emerging from our study is the aberrant localization of the pathogenic 14-3-3γ variants. We find wt 14-3-3γ (with a GFP tag in either N- or C-terminus, also 3xHA tag in the C-terminus) to exhibit mostly cytoplasmic localization, in agreement with previous studies in primary hippocampal neurons [52] and in HCT116 cells [30]. Intriguingly, we observed an aberrant nuclear localization of 14-3-3γ upon mutations in residues R57, R132, and Y133S. Previous studies have reported that other 14-3-3 subtypes (i.e., 14-3-3σ, 14-3-3ζ, and 14-3-3ε) exhibit nucleocytoplasmic shuttling [28,53]. The nuclear export of these 14-3-3 subtypes is, at least partly, Crm1-dependent; for 14-3-3σ, also client protein interaction-dependent [28,53]. Therefore, we hypothesize that the aberrant localization of the 14-3-3γ mutants studied here is related to their strongly reduced ability to bind cytoplasmic partner phosphoproteins and thus be retained in the cytoplasm. Importantly, the nuclear accumulation of mutants 14-3-3γ also suggests a possible neomorphic feature of the mutant proteins, in addition to the dominant negative mechanism of action, which is another interesting future line of investigation to better understand the molecular etiology of *YWHAG*-related disorders.

In this study, we developed a novel in vitro BRET assay to evaluate the ability of 14-3-3γ to bind partner phosphopeptides with higher sensitivity compared to previous analytical tools [14,15,16]. Using our novel assay, we found that the phosphopeptide’s affinities to 14-3-3γ, from the lowest affinity (i.e., pTH) to the highest affinity (i.e., ppLRRK2), correlate with the previously published measurements [14,15,16]. Our assay is sensitive to the external modulator (R18 peptide inhibitor) and permitted us to build a robust HTS platform to find small molecules modulating the binding between 14-3-3γ-R57C and ppLRRK2. Among the screened FDA-approved drugs, we identified nafamostat mesylate, degarelix, oritavancin diphosphate, and aviptadil acetate as the hits partially restoring the deficient interaction of 14-3-3γ-R57C and the phosphopeptide. Nafamostat mesylate (and its orally available analog sepimostat) is a serine protease inhibitor clinically used as an anticoagulant [54]. Nafamostat has poor oral bioavailability and hence is administered intravenously [55], with plasma half-life in human ranging between 23 and 122 min [56]. Degarelix is a semi-synthetic peptide that acts as an antagonist of the gonadotropin-releasing hormone (GnRH) receptor, suppressing testosterone production and used in the treatment of prostate cancer [57]. Upon subcutaneous administration, degarelix forms a depot from which it is released slowly, resulting in a half-life of 28–43 days [58]. Oritavancin diphosphate is a semisynthetic lipoglycopeptide antibiotic, with inhibition of peptidoglycan synthesis as its main mechanism of action against Gram-positive bacteria [59]. Oritavancin is administered as a single intravenous dose of 1200 mg with a half-life of 16.3 days [60]. Aviptadil acetate is a synthetic form of human vasoactive intestinal peptide that exerts anti-inflammatory activities, especially in the lung and has been used to treat inflammatory conditions such as asthma and acute respiratory distress syndrome [61,62]. Aviptadil is administered intranasal or intravenously and has a very short plasma half-life of 1–2 min [63].

Among these drugs, only nafamostat and oritavancin are expected to be cell-permeable. Nafamostat is known to target intracellular signaling pathways, such as NF-κB [64], while oritavancin has been reported to accumulate to high levels in lysosomes of eukaryotic cells [65]. On the other hand, degarelix and aviptadil, which are respectively semisynthetic and synthetic peptides, target GPCRs on the cell surface and thus far no literature reported their abilities to penetrate the cell membrane. These factors should be considered for future studies aiming to evaluate the efficacy of these four drugs on increasing the binding of 14-3-3γ and partner phosphoproteins in cells and in vivo.

An important factor to consider further is whether these drugs are capable of crossing the blood–brain barrier (BBB) to exert their efficacy in neurological patients. A study on a rat model showed that intravenous application of nafamostat mesylate exerts neuroprotective and anti-inflammatory effects on cerebral ischemia through thrombin inhibition in neurons, indicating the capacity of the drug to cross BBB [66]. Nafamostat is also reported to improve neurological outcomes and preserve neuronal axons and dendrites in a chronic ischaemic stroke model rat, suggesting that nafamostat promotes axonal regeneration [67]. In a rabbit model of meningitis, oritavancin (administered intravenously) showed limited (1–5%) penetration to cerebrospinal fluid [68], and was efficacious to treat pneumococcal meningitis [68,69]. However, these studies must be taken with caution as both ischemia and meningitis might cause BBB disruption and increase its permeability [70,71]. Furthermore, the neuroprotective effect of nafamostat and the CNS antimicrobial effect of oritavancin remain to be proven in humans. Thus far, no study has reported the BBB penetration of degarelix and aviptadil acetate. Altogether, quantitative measurements on the brain bioavailability of these drugs in healthy animal models are needed to assess their potency to be developed as drugs for neurological diseases, including *YWHAG*-related disorders.

To expand the possibility of finding a treatment for *YWHAG*-related disorders, future HTS using our in vitro BRET assay can be performed using a larger drug library, such as the ReFRAME collection (12,000 compounds that have reached clinical development or undergone significant preclinical profiling) [72]. Our in vitro BRET assay can also be applicable to screen for modulators (enhancers or inhibitors) of the interaction between wt 14-3-3γ (and other 14-3-3 proteins) with their respective phosphopeptide clients, for other disease indications than *YWHAG*-related disorders. Indeed, members of the 14-3-3 protein family have been shown to be upregulated in several types of cancer and to promote cancer progression, which is the case, e.g., for 14-3-3γ in colorectal cancer [73] or 14-3-3ζ in non-small cell lung carcinoma [74] and hepatocellular carcinoma [75]. A significant number of hits identified in our HTS (and not pursued further), indeed, led to the decreased binding of wt 14-3-3γ to the phosphopeptide. The crucial roles of 14-3-3 proteins in other neurological and neurodegenerative disorders might also benefit from our robust screening platform. As described earlier, LRRK2 variants are the most common genetic cause of PD, where these variants lose their interaction with 14-3-3 proteins [34,50]. Thus, our assay can be tailored to screen for compounds enhancing the interaction between a pathogenic variant of LRRK2 and wild-type 14-3-3.

## 5. Conclusions

We characterized the molecular properties of the most common pathogenic variants of 14-3-3γ causing *YWHAG*-related disorders. Variants R57C, R57G, R132, and Y133S showed diminished capability to bind partner phosphopeptides in vitro and phosphoproteins in cells. Unexpectedly, we also discovered atypical nuclear accumulation of the mutant proteins. Using a novel in vitro BRET assay, we established a screening platform that resulted in the discovery of four FDA-approved drugs able to recover the interaction between the R57C mutant and the phosphotargets. Future studies aiming at the validation of these hits in an animal model of *YWHAG*-related disorders could deliver a novel targeted treatment to this rare yet devastating pediatric neurological disorder. Altogether, our study highlights the high potential of the rare disease-directed drug discovery workflow we established previously [41], arguing for the scaling up of our workflow to other rare diseases.

## Figures and Tables

**Table 1:** List of peptides used in this study.

Peptide	Peptide Sequence ^1^	Reference
FITC-pTH	MPTPDATTPQAKGFRRAV**pS**ELDAKQAEAIMSPRFIGRRQSLIE	[14]
FITC-ppLRRK2	QRHSN**pS**LGPIFDGSGGGSGIKARAS**pS**SPVILVGTHLD	[15]
FITC-pSLP76	FPQSA**pS**LPPYFS	[16]
R18 peptide	PHCVPRDLSWLDLEANMCLP	[17]

^1^ Phosphorylated serine is highlighted in bold.

**Table 2:** List of plasmids used in this study.

Target Plasmid	Source Plasmid	Linearization Sites	Primers for Fragment Amplification
14-3-3γ-GFP or 14-3-3γ-HA	pEGFP-N1 or p3xHA-N1	EcoRI	Fwd:CCCGCGGTACCGTCGACTGCAGATTGTTACCCTCACCGCCATCG Rev:CAGATCTCGAGCTCAAGCTTCGATGGTGGACCGTGAACAACTGG
14-3-3γ-Nluc	pET23a_GNAO1 [19,20]	NcoI, EcoRI	Nluc fragment: Fwd:CGATGGCGGTGAGGGTAACAATGGTGGAGGCGGGACGCGTTCTG Rev:CGCAAGCTTGTCGACGGAGCTCGTCACAGAATGCGTTCGCACAGCCGC 14-3-3γ fragment: Fwd:GATCTCACCATCACCATCACCATGTGGACCGTGAACAACTGGTGC Rev: ATTGTTACCCTCACCGCCATCG

**Table 3:** Primers used to generate 14-3-3γ, 3xHA-TH, and GFP-SLP76 mutants.

Plasmid	Primers
14-3-3γ-R57C-GFP and 14-3-3γ-R57C-3xHA	Fwd: GTTGGCGCTCGTTGCAGCTCTTGGCGCGTTATTAGTTCC Rev: GCCAAGAGCTGCAACGAGCGCCAACCACATTCTTATATG
14-3-3γ-R57G-GFP and 14-3-3γ-R57G-3xHA	Fwd: GTTGGCGCTCGTGGCAGCTCTTGGCGCGTTATTAGTTCC Rev: GCCAAGAGCTGCCACGAGCGCCAACCACATTCTTATATG
14-3-3γ-R132C-GFP and 14-3-3γ- R132C-3xHA	Fwd: GGGCGATTATTACTGTTATCTGGCAGAAGTGGCTACCG Rev: TGCCAGATAACAGTAATAATCGCCCTTCATTTTCAGG
14-3-3γ-Y133S-GFP and 14-3-3γ-Y133S-3xHA	Fwd: GATTATTACCGTTCTCTGGCAGAAGTGGCTACCGGTG Rev: ACTTCTGCCAGAGAACGGTAATAATCGCCCTTCATTTTC
3xHA-TH	Fwd: AAGTCCGGAATGCCCACCCCC Rev: TGGGATCCCTAGCCAATGGCA
GFP-SLP76	Fwd: AAGGAATTCATGGCACTGAGG Rev: TGGGATCCGTTGGGTACCCT

## Data Availability

All data are available through main text and Supplementary Materials.

## Supplementary Materials

The following supporting information can be downloaded at: https://www.mdpi.com/article/10.3390/cells14080559/s1, Figure S1: Cellular properties of wild-type 14-3-3γ and the mutant variants; Figure S2: In vitro BRET assay between 14-3-3γ-nLuc and phosphopeptides; Figure S3: Results of the primary screen to find the modulator of 14-3-3γ-R57C with ppLRRK2; Figure S4: In vitro hit validation.
