# Supplementary material for: A Personalized 14-3-3 Disease-Targeting Workflow Yields Repositioning Drug Candidates
Source: Cells. 2025 Apr 8;14(8):559. doi: 10.3390/cells14080559 (PMC12025923; doi:10.3390/cells14080559)
Supplement: Supplementary file 1 [file cells-14-00559-s001.zip › cells-3536419-supplementary.pdf]

Supplementary Figure S1. Cellular properties of wild-type 14-3-3γ and the mutant variants.

(A,B) GFP tagging at either C- or N- terminus of wild-type 14-3-3γ result in similar protein expression level (A) and predominantly cytoplasmic localization (B) in N2a cells. (C,D) Expression of C-terminally tagged wild-type and mutants 14-3-3γ. N2a cells were transfected with wild-type or mutants 14-3-3γ-GFP . Protein expression was then analyzed by SDS–polyacrylamide gel electrophoresis (SDS-PAGE) and Western blot. Antibody against GFP was used for detection of 14-3-3γ and β-actin was used as loading control (C). Quantification of the expression level of different 14-3-3γ constructs (n=4-6) (D). (E) Similar to 14-3-3γ-GFP, wildtype 14-3-3γ-3xHA mostly localizes in cytoplasm, while mutants 14-3-3γ-3xHA show increase nuclear localizations. (F) Mean fluorescence intensity ratios of 14-3-3γ-GFP variants at the cytoplasm versus entire cells (n= 28-30). (G, H) Mutants R57C also show aberrant nuclear localization in human neuroblastoma cells SH-SY5Y. SH-SY5Y cells expressing wild-type 14-3-3γ-GFP or mutant R57C and stained with DAPI in magenta for nuclei. Scale bar: 10 μm (G). Mean fluorescence intensity ratios of 14-3-3γ-GFP variants at the nucleus versus cytoplasm (n= 14-20) (H). Data in panel D, F, and H represent mean ± SEM. Statistical analysis was performed using one sample t-test (panel D), one-way ANOVA followed by Dunnet's multiple comparison test (panel F), or student t-test (panel H). *p<0.05, **p<0.01, ***p<0.001, ****p<0.001.

Supplementary Figure S2. Mutants 14-3-3γ loss their interactions with partner phosphoprotein in vitro and in cells

(A) 1nM of 14-3-3γ-nLuc or nLuc was mixed with increasing concentrations of FITC-pTH (A), FITCppLRRK2 (B), or FITC-pSLP76 (C). While nLuc does not show increase BRET signal in the presence of phosphopeptides, wild-type 14-3-3γ-nLuc exhibited a dose-dependent increase of the signal, indicating the specificity binding of the phosphopeptides to 14-3-3γ. BRET signal from nLuc is then used to subtract BRET signal of 14-3-3γ-nLuc, resulting in the net BRET signal between 14-3-3γ and phosphopeptides as shown in Fig.2C-E. (D) 1nM of wild-type 14-3-3γ-nLuc was mixed with ppLRRK2 (10 nM), pTH (1 μM), and pSLP76 (100 nM) and increasing concentration of R18 peptide before subjected to BRET assay. R18 peptide inhibited phoshopeptides binding to wild-type 14-3-3γ-nLuc. (E,F) 1nM of wild-type or R57C 14-3-3γ-nLuc was mixed DMSO or 100 μM fusicoccin A (FC-A) and varying concentrations of pTH (E) or pLRRK2 (F).Fusicoccin A did not improved pTH or pLRRK2 binding to 14-3-3γ-nLuc. All data represent mean ± SEM (n≥3). (G,H) Cellular interactions of 14-3-3γ with TH or SLP76 as determined by IP in HEK293T cells. HEK293T cells were co-transfected with wild-type or R57C mutant 14-3-3γ-GFP and 3xHA-TH. IP of 14-3-3γ was done using a nanobody against GFP, and the co-precipitation of TH was analyzed by SDS-PAGE and Western blot. Antibody against GFP was used for detection of 14-3-3γ and against HA for TH (G). HEK293T cells were co-transfected with GFP-SLP76 and wild-type or R57C mutant 14-3-3γ-3xHA. IP of SLP76 was done using a nanobody against GFP, and the co-precipitation of 14-3-3γ was analyzed by

SDS-PAGE and Western blot. Antibody against GFP was used for detection of SLP76 and against HA for 14-3-3γ (H).

Supplementary Figure S3. Results of primary screen to find modulator of 14-3-3γ-R57C with ppLRRK2.

Raw data of primary screen using 8 plates of compound from the FDA-approved and pharmacopeial library are presented here. 14-3-3γ-R57C-nLuc (1 nM) was mixed with FITC-ppLRRK2 (100 nM) and compounds (12.5 μM, black dots) before the addition of furimazine (250 nM). BRET signal between wild-type 14-3-3γnLuc and FITC-ppLRRK2 served as positive controls (green dots) and signal between 14-3-3γ-R57C-nLuc, FITC-LRRK2, and DMSO served as negative controls (light blue dots). Additionally, we included BRET signal from nLuc and FITC-LRRK2 in each plate (red dots). Calculated Z' factors for all plates were >0.7, indicating a very robust assay. Primary hit criteria were compounds that modulate BRET signal >± 3SD from the negative controls, as shown by threshold in red lines, resulting in 95 primary hits (dark blue dots).

Supplementary Figure S4. *In vitro* hit validation.

(A, B) 1nM of recombinant proteins was mixed with 100nM FITC-pLRRK2 and increasing concentrations of (A) nafamostat or (B) degarelix before subjected to in vitro BRET assay. Both compounds did not affect nLuc. Nafamostat >3.3 μM significantly affected BRET signal in 14-3-3γ-R57C-nLuc, while maximum BRET signal for degarelix was achieved at concentration of 1 μM. (C, D) The effect of sepimostat (25μM), an oral analog of nafamostat, on the BRET signal between pLRRK2 and wild-type 14-3-3γ-nLuc (C) or 14-3-3γR57C-nLuc (D). (E, F) The effect of aviptadil acetate and oritavancin diphosphate (both 25 μM) on the BRET signal between pLRRK2 and wild-type 14-3-3γ-nLuc (E) or 14-3-3γ-R57C-nLuc (F). All data represent mean ± SD (n=4). Statistical analysis in panel A and B was performed using two-ways ANOVA followed by Dunnett's multiple comparisons test. ***p<0.001, ****p<0.0001.

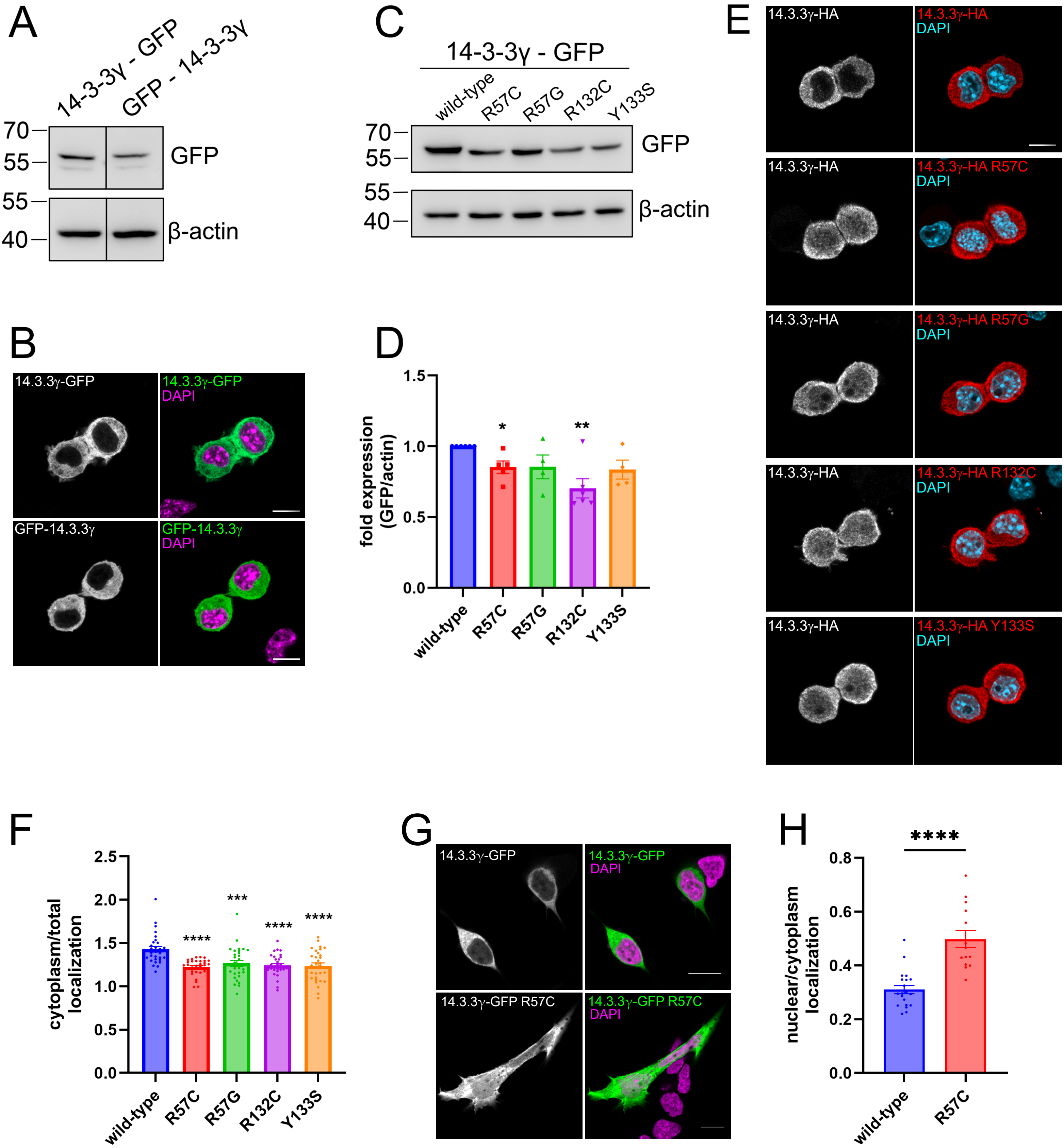

A
14-3-3γ - GFP
GFP - 14-3-3γ
70
55
GFP
55
40
β-actin
B
14.3.3γ-GFP
14.3.3γ-GFP
DAPI
GFP-14.3.3γ
GFP-14.3.3γ
DAPI
C
14-3-3γ - GFP
wild-type
R57C
R57G
R132C
Y133S
GFP
β-actin
D
fold expression (GFP/actin)
1.5
1.0
0.5
0.0
*
**
wild-type
R57C
R57G
R132C
Y133S
E
14.3.3γ-HA
14.3.3γ-HA
DAPI
14.3.3γ-HA R57C
14.3.3γ-HA R57G
14.3.3γ-HA R132C
14.3.3γ-HA Y133S
F
cytoplasm/total localization
2.5
2.0
1.5
1.0
0.5
0.0
****
***
****
****
wild-type
R57C
R57G
R132C
Y133S
G
14.3.3γ-GFP
14.3.3γ-GFP
DAPI
14.3.3γ-GFP R57C
14.3.3γ-GFP R57C
DAPI
H
****
nuclear/cytoplasm localization
0.8
0.6
0.4
0.2
0.0
wild-type
R57C

Figure S1

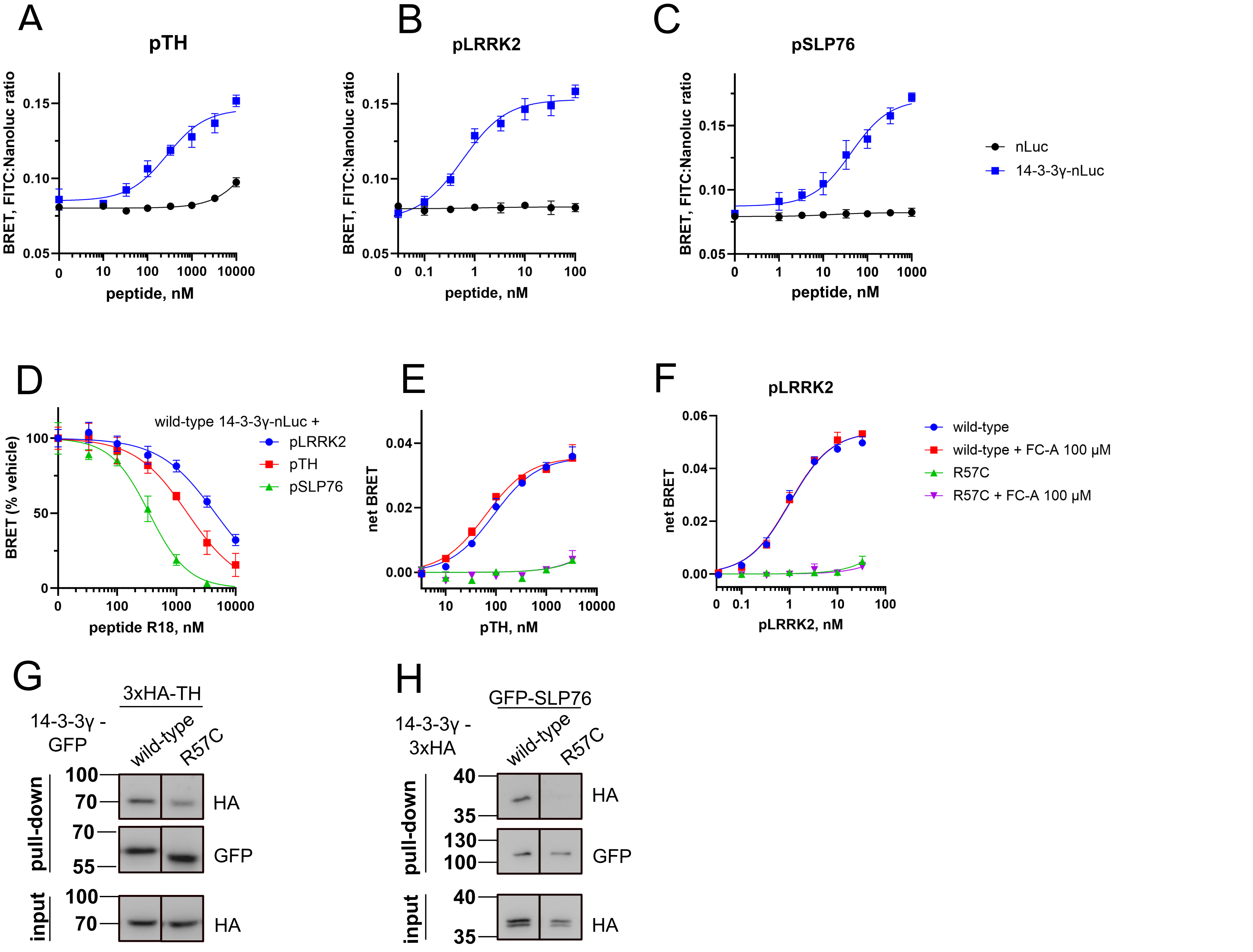

A
pTH
BRET, FITC:Nanoluc ratio
0.15
0.10
0.05
0
10
100
1000
10000
peptide, nM
B
pLRRK2
BRET, FITC:Nanoluc ratio
0.15
0.10
0.05
0
0.1
1
10
100
peptide, nM
C
pSLP76
BRET, FITC:Nanoluc ratio
0.15
0.10
0.05
0
1
10
100
1000
peptide, nM
nLuc
14-3-3γ-nLuc
D
BRET (% vehicle)
100
50
0
wild-type 14-3-3γ-nLuc +
pLRRK2
pTH
pSLP76
0
100
1000
10000
peptide R18, nM
E
net BRET
0.04
0.02
0.00
10
100
1000
10000
pTH, nM
F
pLRRK2
net BRET
0.06
0.04
0.02
0.00
0
0.1
1
10
100
pLRRK2, nM
wild-type
wild-type + FC-A 100 μM
R57C
R57C + FC-A 100 μM
G
3xHA-TH
14-3-3γ - GFP
wild-type
R57C
pull-down
input
100
70
70
55
100
70
HA
GFP
HA
H
GFP-SLP76
14-3-3γ - 3xHA
wild-type
R57C
pull-down
input
40
35
130
100
40
35
HA
GFP
HA

Figure S2

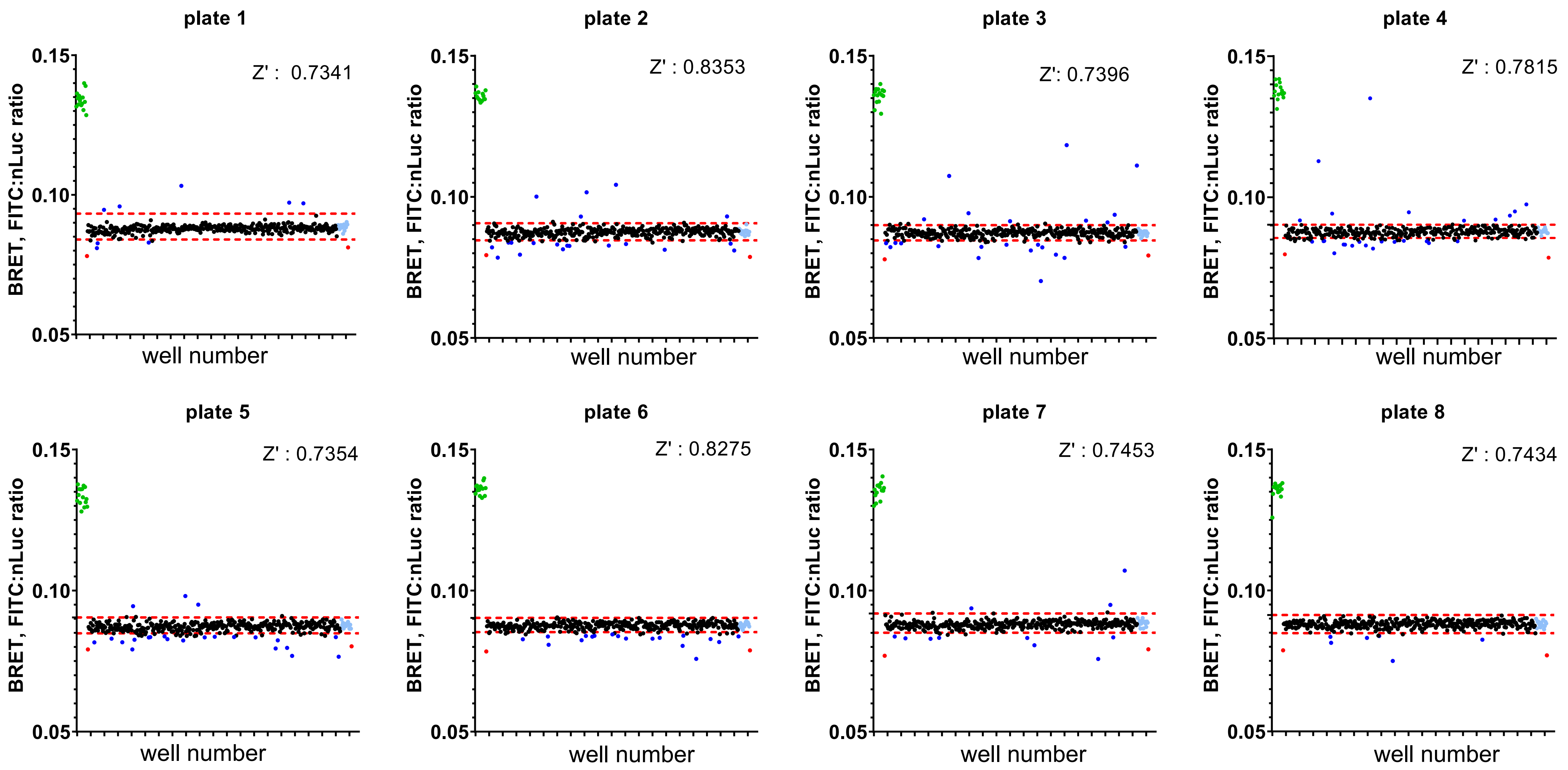
plate 1
Z' : 0.7341
plate 2
Z' : 0.8353
plate 3
Z': 0.7396
plate 4
Z' : 0.7815
plate 5
Z' : 0.7354
plate 6
Z' : 0.8275
plate 7
Z' : 0.7453
plate 8
Z' : 0.7434
BRET, FITC:nLuc ratio
0.15
0.10
0.05
well number

Figure S3

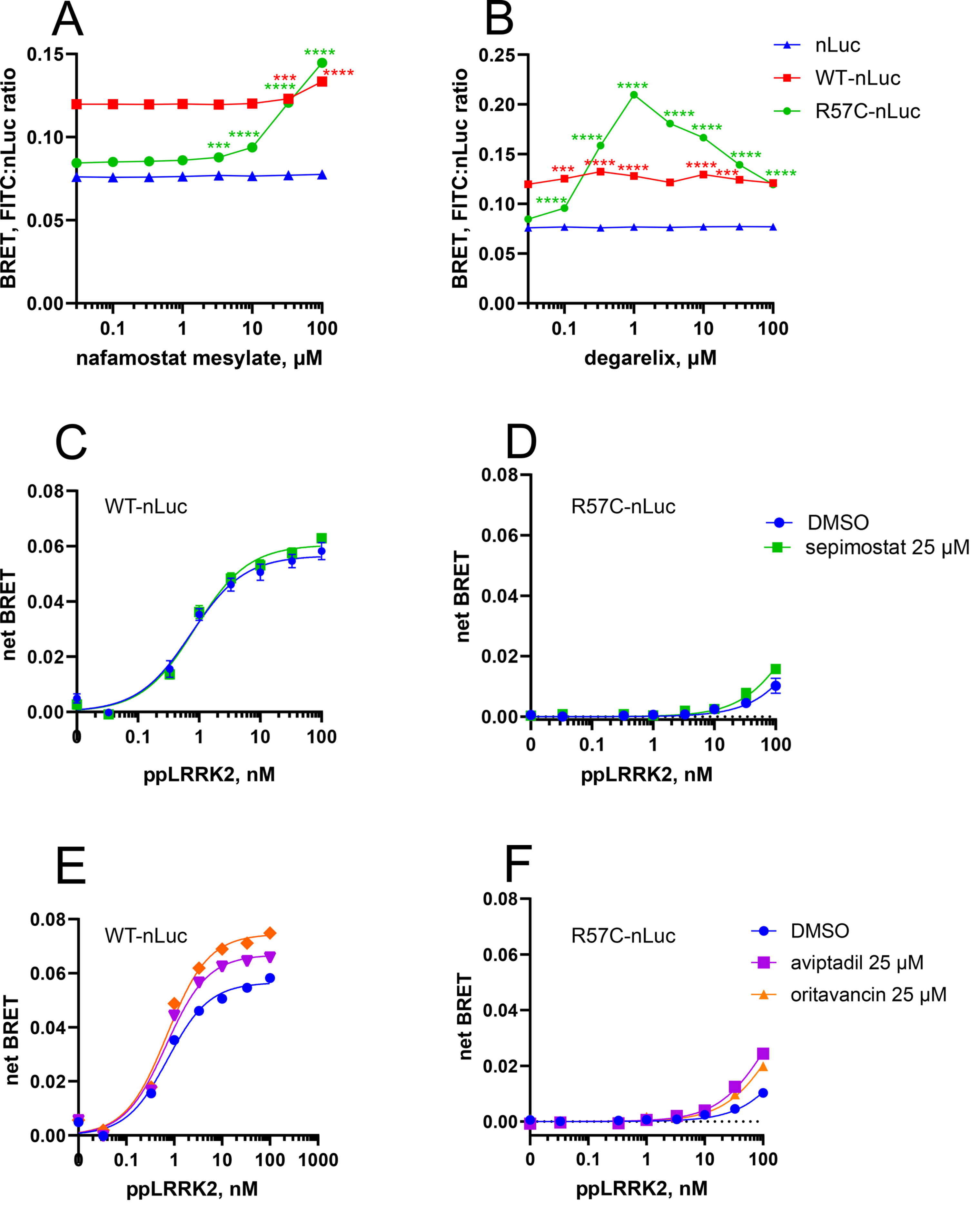
A
B
BRET, FITC:nLuc ratio
nafamostat mesylate, μM
degarelix, μM
nLuc
WT-nLuc
R57C-nLuc
C
D
WT-nLuc
R57C-nLuc
DMSO
sepimostat 25 μM
net BRET
ppLRRK2, nM
E
F
aviptadil 25 μM
oritavancin 25 μM

Figure S4
